# Comparison of risk scoring systems for upper gastrointestinal bleeding in patients after renal transplantation: a retrospective observational study in Hunan, China

**DOI:** 10.1186/s12876-022-02426-3

**Published:** 2022-07-25

**Authors:** Rui Wang, Qiang Wang

**Affiliations:** 1 Department of Gastroenterology, The Third Xiangya Hospital of Central South University, Changsha, 410013 Hunan Province China; 2 Department of Transplantation, The Third Xiangya Hospital of Central South University, Changsha, 410013 Hunan Province China

**Keywords:** Upper gastrointestinal bleeding, Renal transplant recipient, Glasgow Blatchford score, AIMS65, Pre-endoscopy Rockall score

## Abstract

**Background:**

Upper gastrointestinal bleeding (UGIB) is a common complication in renal transplant recipients. However, the risk stratification value of bleeding scoring systems in these patients is unclear, and data regarding risk factors are limited.

**Methods:**

Clinical data of renal transplant recipients in The Third Xiangya hospital were collected. The predictive ability of Glasgow Blatchford score (GBS), pre-endoscopy Rockall score (pRS), and AIMS65 score were assessed by the area under the receiver operating characteristic curve (AUROC). Risk factors of UGIB were analyzed using binary logistic regression analysis.

**Results:**

A total of 220 patients were enrolled, of which 55 with UGIB. Endoscopy improved the overall survival rate of patients. Glasgow Blatchford score (AUROC 0.868) performed best at predicting UGIB patients who need intervention or death, with a threshold of 10, sensitivity and specificity were 82.4% and 70%, respectively. In terms of predicting mortality, the GBS score was comparable with AIMS65 score (*p* = 0.30) and pRS score (*p* = 0.42). Viral hepatitis, intravenous hormone usage, low platelet count, and low albumin level were significant factors associated with UGIB.

**Conclusions:**

The Glasgow Blatchford score (AUROC 0.868) was best at predicting the need for intervention or death. However, their ability to predict mortality was limited, with AUROC less than 0.8. Our study also identified four independent risk factors for renal transplant recipients with UGIB.

**Supplementary Information:**

The online version contains supplementary material available at 10.1186/s12876-022-02426-3.

## Introduction

Renal transplantation is the best treatment for patients with end-stage kidney disease. However, various postoperative complications lead to frequent readmission and high treatment costs. With increasing numbers of renal transplant recipients, more attention needs to be paid to the risk of post-transplant complications to optimize management. Upper gastrointestinal bleeding (UGIB) is a common complication in renal transplant recipients, with an incidence of 0.3% to 21.5% [1, 2]. Severe UGIB may lead to renal insufficiency and death [3–6]. Possible pathogenesis is excessive gastric acid secretion, inhibition of platelet factor III caused by azotemia, thrombocytopenia, and antiplatelet effect of immunosuppressive drugs, etc. [7, 8] Graft function loss and graft rejection are two risk factors, as shown by several studies [8–11]. However, the study subjects include patients with end-stage kidney disease and some with small sample size. The effectiveness of the data is limited. The issue in patients without renal failure hasn’t been addressed so far.

Early identification and active intervention of critical patients with UGIB play an essential role in improving the prognosis of patients. The international guideline recommends bleeding scoring systems for risk stratification [12]. The full Rockall score and Progetto Nazionale emorragia digestiva (PNED) score need endoscopy before scoring [13, 14], while it may take hours, days, and sometimes weeks for patients to take endoscopy. These scoring systems may delay the risk assessment. Therefore, more and more people are interested in pre-endoscopy risk scores such as Glasgow Blatchford score (GBS), pre-endoscopy Rockall score (pRS), and AIMS65 score [15–17], which can be easily calculated shortly after a patient's presentation. A recent large-scale, multicenter and prospective study found that GBS was best in predicting patients' need for treatment or death, the PNED and AIMS65 scores better predict mortality. Cut-off values can be used to identify high-risk groups[18]. Therefore, the above scoring systems are practical in clinical application.

The scoring system like GBS consists of a patient's medical history, vital signs, and laboratory indicators at admission, in which Different levels of hemoglobin, blood urea nitrogen (BUN), and blood pressure correspond to different scores. The reference values are formulated for the general population. Renal transplant recipients are usually complicated with anemia, azotemia, and hypertension, leading to higher bleeding scores, and being classified as high-risk population. Whether the pre-endoscopy scoring system can be used for these patients is unclear.

This study aims to identify the risk factors of UGIB in renal transplant recipients without renal failure, and to compare the predictive value of the three pre-endoscopy scoring systems for risk stratification. This might facilitate a low-risk group for outpatient management, identify the high-risk group and formulate intervention strategies as soon as possible. Therefore, improving the overall prognosis in two aspects of prevention and intervention.

## Materials and methods

1. Patient selection and data collection: the clinical data of renal transplant recipients in The Third Xiangya Hospital of Central South University from January 2015 to December 2019 were collected for retrospective analysis. The exclusion criteria include patients with lower gastrointestinal bleeding, patients with renal graft failure and dialysis, and patients with gastrointestinal bleeding due to other diseases during hospitalization. A total of 220 patients were enrolled, of which 165 patients were without bleeding and 55 patients with UGIB. The data included gender, age, height, weight, complications (pulmonary infection, diabetes, viral hepatitis), drug use (antithrombotic and anticoagulant drugs, immunosuppressive agents), clinical manifestations, vital signs, and laboratory results of the bleeding (international normalized ratio, platelet, hemoglobin, urea nitrogen, creatinine, etc.), blood transfusion, endoscopy and intervention, surgery or interventional therapy, and patients’ outcomes.

The scores of all patients before endoscopy on the first day of admission were calculated, and risk stratification was carried out by using pRS, GBS, and AIMS65 score. It is worth noting that risk stratification was carried out before endoscopy, therefore, standards related to endoscopy in the pRS were not included, leading to the total score of the pRS between zero to seven.

UGIB is defined as the clinical manifestation of hematemesis and/or black stool. Endoscopic reports describing active bleeding (i.e. variceal veins, ulcers, mucosal lesions, etc.) or recent bleeding (i.e. adhesive clots), are defined as diagnostic endoscopy. An endoscopic report not identifying bleeding sources is defined as non-diagnostic endoscopy. Patients who need urgent hemostatic intervention are defined as having a blood transfusion, or surgery, or non-surgery treatment (namely endoscopy and radiological intervention) to stabilize the vital sign.

The endpoints were composite outcomes (the need for urgent hemostatic intervention or death), the need for endoscopic intervention, and 90-day mortality. Death was determined through medical records in hospital healthcare systems and confirmed with patients’ relatives if necessary.

2. Statistical analysis: SPSS 22.0 software was used for data analysis. The measurement data were expressed as mean ± standard deviation or median ± interquartile range, and percentages were calculated for categorical variables. The comparison between the two groups was conducted by independent sample t-test. The counting data were expressed by the number of cases and percentage, and the chi-square test was used for the comparison between the two groups. Binary logistic regression analysis (forward: likelihood ratio (LR)) was used to analyze the independent risk factors of UGIB, including variables with a p-value less than 0.05 at group comparison in the single factor analysis. The survival curve was drawn by the Kaplan Meier method and compared between the two groups (*p* < 0.05). The area under the receiver operating characteristic curve (AUROC) was drawn to evaluate the predictive ability of each scoring system. For each scoring system, the effective cut-off value was obtained by calculating the Youden index. De Long's index was used to compare AUROC values. Sensitivity, specificity, negative predictive value (NPV), and positive predictive value (PPV) for study outcomes were calculated.

## Results

### Clinical characteristics of UGIB

There were 55 patients with UGIB and 165 patients without UGIB enrolled. In UGIB patients, the mean time to bleeding was 54.16 ± 55.89 months after transplantation. 29.1% died within 3 months after UGIB, with an average survival time of 1.42 ± 1.30 months after bleeding. There were 40 patients with black stool (72.7%), 15 patients with hematemesis and black stool (27.3%). Endoscopy has revealed the bleeding source in 22 patients, accounting for 66.7% of all 33 patients who underwent endoscopy (Table 1 and Additional file 1: Table 1). Ulcer was the most common cause, of which five patients received endoscopic treatment.

**Table 1:** Clinical characteristics, therapeutic yields, and outcomes of renal transplant recipients with upper gastrointestinal bleeding

Characteristics	Data
Time after transplantation (Months)	54.16 ± 55.89
eGFR (before bleeding, ml/min/1.73m^2^)	48.35 ± 26.95
eGFR (after bleeding, ml/min/1.73m^2^)	42.38 ± 41.33
Systolic blood pressure (mmHg)	128 ± 24
Heart rate (/min)	92 ± 19
Hemoglobin before bleeding (g/ml)	111.46 ± 29.9
Hemoglobin after bleeding (g/ml)	80.7 ± 35.8
Melena, n (%)	40 (72.7%)
Haematemesis and melena, n (%)	15 (27.3%)
*Gastroscopic yields*	
Nothing abnormal, n (%)	11 (20.0%)
Erosive disease, n (%)	4 (7.3%)
Gastric/duodenal ulcer, n (%)	16 (29.1%)
Variceal bleeding, n (%)	2 (3.6%)
*Treatment*	
Gastroscopic treatment, n (%)	5 (9.1%)
Surgery/radiological intervention, n (%)	1 (1.8%)
Without gastroscopy, n (%)	22 (40.0%)
*Outcomes*	
Mortality, n (%)	16 (29.3%)
*Average score*	
Glasgow Blatchford score, n (%)	10.2 ± 4.2
AIMS65 score, n (%)	0.3 ± 0.6
Pre-endoscopy Rockall score, n (%)	1.2 ± 1.3

eGFR: estimated glomerular filtration rate

Estimated glomerular filtration rate (eGFR) in both eGFR ≥ 60 ml / (min * 1.73m^2^) group and eGFR < 60 ml / (min * 1.73m^2^) group decreased after UGIB. There was no difference in survival rate between the two groups (*p* = 0.48). There was no significant difference in renal function between patients with or without endoscopy. However, the overall survival rate of those with endoscopy was significantly improved (HR = 4.30, 95%CI: 1.51–12.26, *p* < 0.01) (Additional file 1: Fig. 1).

**Fig. 1:**
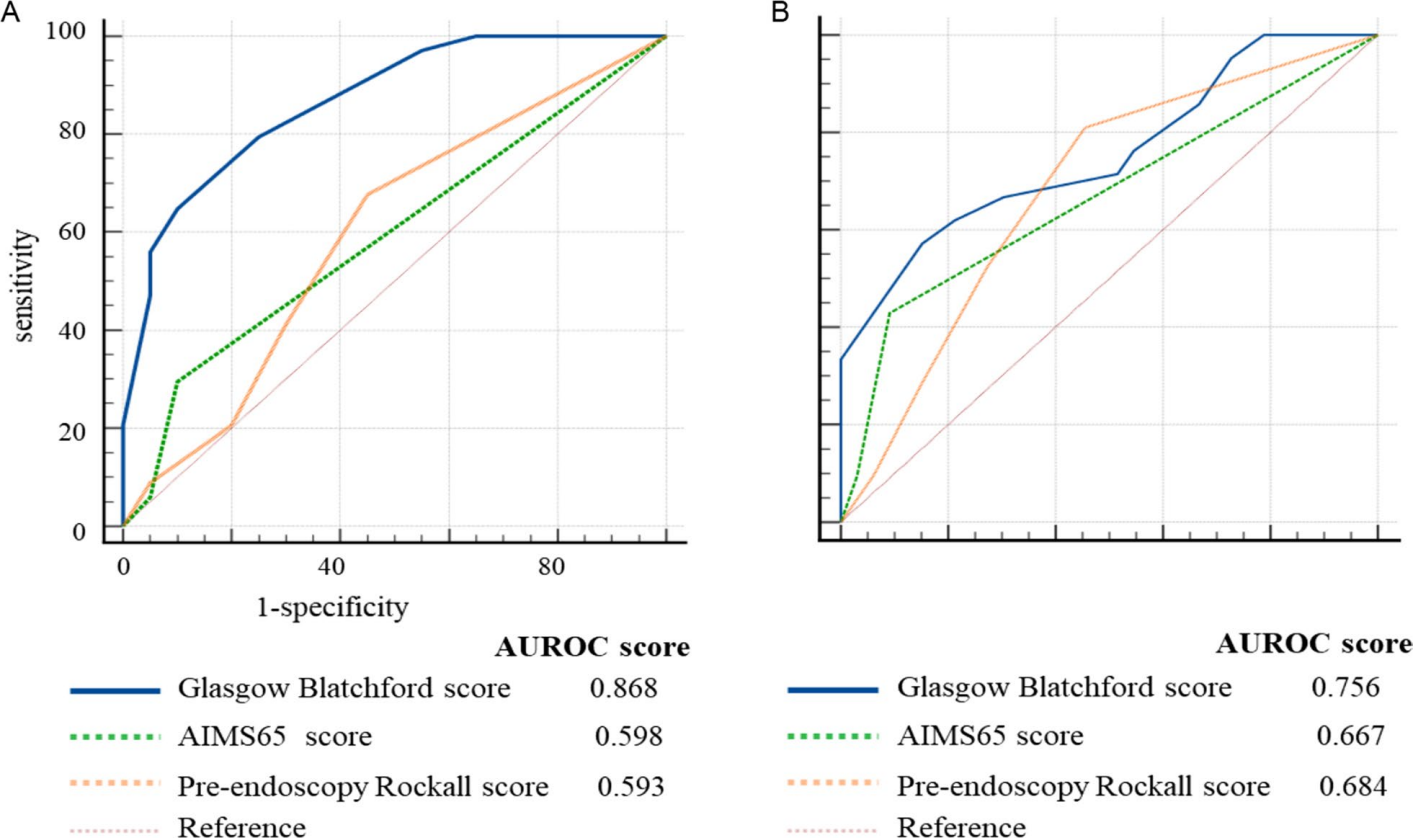
**A** Comparison of three pre-endoscopy scoring systems on predicting need for intervention or death. **B** Comparison of three pre-endoscopy scoring systems on predicting 90-day mortality. AUROC: area under the receiver operating characteristic curve

### Comparison of scores’ ability in predicting different outcomes for renal transplant recipients with UGIB

We have compared the power of different scoring systems for risk stratification in renal recipients with UGIB. Overall, the average score of GBS was 10.2 ± 4.2 points, AIMS65 was 0.3 ± 0.6 points, and pRS was 1.2 ± 1.3 points, as shown in Table 1.

### Intervention or mortality

A total of 35 patients (63.6%) required for urgent hemostatic intervention or died, of which 29 patients received a blood transfusion, 5 patients with ulcer bleeding were treated under gastroscopy, and 1 patient with gastric variceal bleeding underwent surgery. In predicting need for urgent intervention or death, the ability of GBS (AUROC 0.868) was significantly higher than AIMS65 (AUROC 0.598, *p* < 0.001) and pRS (AUROC 0.593, *p* < 0.001). There was no significant difference between AIMS65 and pRS (*p* = 0.95) (Table 2 and Fig. 1A). Table 3 shows the power of the three scoring systems to predict high-risk patients who needed urgent hemostatic intervention or might die at optimal cut-offs. A Glasgow Blatchford score of 10 or more was best at predicting the composite endpoint, with a sensitivity of 82.4%, specificity of 70%, a positive predictive value of 82.7%, and a negative predictive value of 70%. However, they were all poor at predicting low-risk populations who might not need urgent hemostatic intervention and survive at optimal thresholds (Additional file 1: Table 2). With a threshold GBS of 6 or less, the specificity reached 97.1%, but the sensitivity was only 45.0%. Urgent hemostatic intervention or death was recorded in 1.8% of the patients with GBS of 6 or less, while 70.9% of the patients with AIMS65 of 0 or less, and 27.3% of the patients with pRS of 0 or less.

**Table 2:** Discriminative ability of evaluating scoring system

Outcome by scoring system	AUROC
*Intervention or death*	
Glasgow blatchford score	0.868
AIMS65 score	0.593
Pre-endoscopy Rockall score	0.598
*Urgent endoscopic intervention*	
Glasgow blatchford score	0.637
AIMS65 score	0.522
Pre-endoscopy rockall score	0.552
*Mortality*	
Glasgow blatchford score	0.756
AIMS65 score	0.667
Pre-endoscopy rockall score	0.684

**Table 3:** Optimal cut-off values for scoring system to predict patients who need intervention or might die

Scoring system	Cut-off	No (%) of patients classified as high risk	Sensitivity (%)	Specificity (%)	PPV (%)	NPV (%)
Glasgow blatchford	≥ 10	35 (63.6%)	82.4	70	82.7	70
AIMS65	≥ 1	12 (21.8%)	28.6	90	83.3	41.9
Pre-endoscopy Rockall score	≥ 1	32 (58.2%)	65.7	55	71.9	33.3

PPV = positive predictive value; NPV = negative predictive value

### Urgent endoscopic intervention

Endoscopic intervention helps to stabilize vital signs. Therefore, we analyzed the predictive ability of the three pre-endoscopy scoring systems in identifying patients requiring urgent endoscopic intervention. Both AIMS65 (AUROC 0.522) and pRS (AUROC 0.552) failed at predicting it (Table 2 and Additional file 1: Fig. 2). GBS (AUROC 0.637) had a poor predictive ability, with a threshold of 8 or more, sensitivity, specificity, positive predictive value and negative predictive value were 100%, 33.3%, 23%, and 100% respectively.

### Mortality

A total of 16 (29.3%) patients died within 90 days after UGIB. In predicting mortality, the ability of GBS (AUROC 0.756) was comparable to AIMS65 (AUROC 0.667, *p* = 0.30) and pRS (AUROC 0.684, *p* = 0.42), the same with AIMS65 and pRS (*p* = 0.85) (Table 2 and Fig. 1B). With a GBS threshold of 14 or more, sensitivity, specificity, positive predictive value, and negative predictive value were 68.8%, 84.6%, 64.7%, and 66.7% respectively (Additional file 1: Table 3). While with an optimal threshold of 1 or more, AIMS65 and pRS showed either low sensitivity or specificity. Therefore, all three of them had limited value in predicting mortality.

### Risk factors for UGIB after renal transplantation

Age, intravenous hormone usage, hemoglobin, platelet, international normalized ratio, urea nitrogen, creatinine, estimated Glomerular Filtration Rate, albumin, complications with viral hepatitis and cardio-cerebrovascular disease were significant factors associated with UGIB (Table 4). Binary logistic regression analysis showed that viral hepatitis (OR: 9.468,95% CI: 2.277–39.396), intravenous hormone methylprednisolone usage (OR: 3.560,95% CI: 1.335–9.490), low platelet count (OR: 0.994,95% CI: 0.988–0.999) and albumin level (OR: 0.835,95% CI: 0.767–0.910) were independent risk factors (Table 5).

**Table 4:** Baseline characteristics in patients with and without upper gastrointestinal bleeding

Characteristics		GIB (n = 55)	No GIB (n = 165)	*p*-value
Sex, n (%)	Male	41 (74.5%)	104 (63.0%)	0.12
Age, average ± SD		47.31 ± 10.45	43.83 ± 10.62	0.04
BMI, kg/m^2^		22.40 ± 3.68	21.61 ± 3.16	0.13
*Systolic blood*				
Pressure, mmHg		128 ± 24	127 ± 13	0.72
Heart rate (/min)		92 ± 19	87 ± 15	0.16
Anticoagulant drugs usage, n (%)	Negative	49 (89.1%)	158 (95.8%)	0.07
Intravenous hormone usage,n (%)	Negative	36 (65.5%)	147 (89.1%)	< 0.001
Hemoglobin, g/L		111.46 ± 29.85	120.69 ± 24.73	0.03
Platelet, 10^9^/L		179.98 ± 84.18	201.60 ± 59.28	0.04
INR		1.06 ± 0.25	0.98 ± 0.13	< 0.001
Urea nitrogen, mmol/L		13.04 ± 8.42	9.91 ± 6.14	< 0.001
Creatinine,μmol/L		170.89 ± 80.33	142.01 ± 87.85	< 0.001
eGFR, ml / (min * 1.73m^2^)		48.35 ± 26.95	58.31 ± 28.13	< 0.001
Albumin, g/L		34.48 ± 5.25	40.00 ± 5.52	< 0.001
Calcineurin inhibitors, n (%)	Tacrolimus	47 (85.5%)	153 (92.7%)	0.10
	Cyclosporine A	8 (14.5%)	12 (7.3%)	
*Complication*				
Diabetes, n (%)	No	47 (85.5%)	155 (93.9%)	0.05
Viral hepatitis (HBV/HCV), n (%)	Negative	43 (78.2%)	160 (97.0%)	< 0.001
Pulmonary infection, n (%)	Negative	34 (61.8%)	116 (70.3%)	0.24
Cardio-cerebrovascular disease, n (%)	Negative	48 (87.3%)	161 (97.6%)	< 0.001
Malignant tumor, n (%)	Negative	52 (94.5%)	160 (97.0%)	0.41
Outcome, n (%)	Mortality	16 (29.1%)	9 (5.5%)	0.03

INR: International normalized ratio; eGFR: estimated glomerular filtration rate; BMI: Body mass index. HBV: hepatitis B virus; HCV: hepatitis C virus

**Table 5:** The independent risk factors of upper gastrointestinal bleeding in patients after renal transplantation

	OR (95%CI)	*p-*value
Viral hepatitis	9.468 (2.277–39.396)	< 0.01
Intravenous hormone usage	3.560 (1.335–9.490)	0.01
Platelet counts	0.994 (0.988–0.999)	0.03
Albumin	0.835 (0.767–0.910)	< 0.001

OR: Odds ratio

## Discussion

UGIB is a common complication in patients after kidney transplantation, leading to renal dysfunction and death in severe cases. Data regarding risk factors are limited, especially in patients without renal failure. Risk stratification value of bleeding scoring system in these patients is unclear. This study found that viral hepatitis infection, intravenous hormone usage, low platelet count, and low albumin level were independent risk factors for UGIB in these patients. A comparison of the three pre-endoscopy scoring systems revealed that the Glasgow Blatchford Score (GBS) performed best in predicting urgent hemostatic intervention or death. A GBS threshold of 10 or more was best at predicting the high-risk patients. Whereas all three had limited value in predicting the need for urgent endoscopy or 90-day mortality. This study provides data on the early identification of patients with bleeding tendency and risk stratification of bleeding severity.

Early identification of high-risk patients needing urgent intervention or at high risk of death can help to improve the prognosis. So far there is no unified scoring system to predict all these endpoints accurately. This study compared the predictive value of three widely used pre-endoscopy scoring systems[17, 19] in renal transplant patients with UGIB. GBS (AUROC 0.868) performed best in predicting the need for urgent intervention or death, which was consistent with Stanley and his colleagues’ study in the general population [18]. The optimal cut-off was 10 for predicting high-risk patients, higher than the threshold 7 in the general population. This might be related to the difference in the biochemical index, the major component of scoring systems. The GBS threshold of 10 might be more accurate for renal transplant recipients.

Identification of a low-risk population was helpful for outpatient management[20]. Stanley and his colleagues[18] indicated that GBS threshold of 1 or less in identifying low-risk patients had a sensitivity of 98.6%, specificity of 34.6%, with endoscopic treatment performed in 1.4%. Some international guidelines also recommend outpatient management of patients with a cut-off less than or equal to 1[21, 22]. We found the three scoring systems were al poor in predicting low-risk patients. For example, with AIMS65 cut-off of 0 or less, 9.1% of patients received endoscopic intervention and 14.5% died. Therefore, some high-risk patients could be incorrectly classified as low-risk patients. However, the GBS scoring system may guide the allocation of clinical beds [23]. Non-monitored ordinary beds could work for patients with a score of 6 or less. For patients with a score of 6 to 10, more observation might be needed. Patients with a score of more than 10 could be recommended for intensive care.

Endoscopy is an essential method for UGIB. To the best of our knowledge, there is no study on the role of endoscopy in renal transplant recipients with UGIB. Endoscopy has limited diagnosis and treatment effects in GIB patients with continuous flow-left ventricular assist devices (CF-LVADs), since it rarely identifies single treatable bleeding source and cannot reduce the recurrence rate of GIB [24]. Our research showed that endoscopy improved the survival rate in renal transplant recipients with UGIB. There was no difference in complications and drug usage between the endoscopy and non-endoscopy groups, therefore, the result is not biased due to these factors (as shown in Additional file 1: Table 1).

However, none of those three scoring systems can effectively predict needing for endoscopic intervention, with AUROC all lower than 0.8, which is consistent with several other studies[17, 25].

The risk of UGIB in renal transplantation would exist for a long time. Our results showed a mortality of 29.3% and graft loss of 32.6% in renal transplant recipients with UGIB, with an obvious drop in recent years. This may be related to a better understanding of UGIB in recent years, more standardized clinical management, and a close follow-up of experienced teams. Several studies[26], including a study of more than 3000 patients[18], have shown that AIMS65 was best at predicting mortality, with mostly AUROC lower than 0.8[27, 28]. In our data, AIMS65 (AUROC 0.667) was no better than GBS (AUROC 0.756) or pRS (AUROC 0.684). The reason might be AIMS65 mainly evaluates patients’ admission situations, but complications other than UGIB might cause death to renal transplant recipients during hospitalization. pRS and GBS scoring systems include other complications, making them more powerful in predicting mortality.

So far, there are limited data on the risk factors of UGIB in these patients. We identified four independent risk factors. A study by Ardalan and his colleagues[29] suggested that intravenous administration of hormones and cytomegalovirus (CMV) infection were risk factors. In our study, CMV infection could not be assessed due to the large time span of our data. With renal allograft failure enrolled, two studies indicated that renal allograft failure was also an independent risk factor[8, 9]. Our study found that the estimated glomerular filtration rate (eGFR), which reflected renal function, was statistically lower in the UGIB group. However, eGFR was not identified as an independent risk factor in our study might for two reasons. First, with average eGFR between 30–59 ml/ (min * 1.73m^2^) in both groups, the renal function staging was both in chronic kidney disease stage 3. Therefore, the bleeding tendency might be at the same level, as shown in a study by Ishigami and his colleagues[30]. Secondly, our study excluded patients with renal allograft failure, which might also lead to the difference.

The main strengths of this study lie in two points. First, some new independent risk factors of UGIB in renal transplant recipients was identified. Second, it compared the predictive ability of three pre-endoscopy bleeding scoring systems in these patients for the first time. However, this study also has some limitations. First, this is a single centred retrospective study. Data collection and risk assessment are determined through existing clinical records, and the sample size is relatively small. Second, although we follow the established clinical treatment guidelines for the choice of treatment methods, the results may vary according to experienced endoscopists and intensive care ability. Third, the period of data collection in this study is extensive. The detailed past histories such as Helicobacter pylori infection, previous peptic ulcer disease and CMV infection were missing and weren’t included in the study.

## Conclusions

In conclusion, we have identified four independent risk factors of UGIB after renal transplantation, including intravenous hormone usage, low platelet count, low albumin level and viral hepatitis. Glasgow Blatchford score is the best to predict intervention or death. The three pre-endoscopy scoring systems have limited value in predicting mortality and the need for endoscopic intervention. However, endoscopy can improve patients' overall survival rate. We recommend endoscopy for UGIB patients without contraindications. Overall, this study provides some new information to improve the prognosis of renal transplant recipients with UGIB from three aspects, prevention, risk stratification and treatment.

## Supplementary Information

**Additional file 1: Supplementary Table 1.** Clinical characteristics of renal transplant recipients with upper gastrointestinal bleeding between endoscopic and non-endoscopic groups. **Supplementary Table 2.** Optimal cut-off values of each scoring system in predicting patients who might not need intervention. **Supplementary Table 3.** Optimal thresholds to predict 90-day mortality in renal transplant recipients with upper gastrointestinal bleeding. **Supplementary figure 1.** The survival rate and renal function of kidney transplant recipients with upper gastrointestinal bleeding. **Supplementary figure 2.** Comparison of three pre-endoscopy scoring systems on predicting need for urgent endoscopic intervention. *AUROC*: area under the receiver operating characteristic curve.

## Data Availability

All data generated or analysed during this study are included in the manuscript and its supplementary information files, which is also uploaded in respective section. The raw datasets generated and/or analyzed during the current study are not publicly available due to protecting patients’ identity, but are available from the corresponding author on reasonable request.

## Abbreviations

UGIB: Upper gastrointestinal bleeding
AUROC: Area under the receiver operating characteristic curve
CMV: Cytomegalovirus
CF-LVADs: Continuous flow-left ventricular assist devices
eGFR: Estimated glomerular filtration rate
GBS: Glasgow Blatchford score
INR: International standardized ratio
NPV: Negative predictive value
PNEDscore: Progetto Nazionale emoragia digest score
PPV: Positive predictive value
pRS: Pre-endoscopy Rockall score
