# Supplementary material for: Comparison of risk scoring systems for upper gastrointestinal bleeding in patients after renal transplantation: a retrospective observational study in Hunan, China
Source: BMC Gastroenterol. 2022 Jul 25;22:353. doi: 10.1186/s12876-022-02426-3 (PMC9316734; doi:10.1186/s12876-022-02426-3)
Supplement: Supplementary file 1 — Additional file 1: Supplementary Table 1. Clinical characteristics of renal transplant recipients with upper gastrointestinal bleeding between endoscopic and non-endoscopic groups. Supplementary Table 2. Optimal cut-off values of each scoring system in predicting patients who might not need intervention. Supplementary Table 3. Optimal thresholds to predict 90-day mortality in renal transplant recipients with upper gastrointestinal bleeding. Supplementary figure 1. The survival rate and renal function of kidney transplant recipients with upper gastrointestinal bleeding. Supplementary figure 2. Comparison of three pre-endoscopy scoring systems on predicting need for urgent endoscopic intervention. AUROC: area under the receiver operating characteristic curve. [file 12876_2022_2426_MOESM1_ESM.docx]

Supplementary Table 1. Clinical characteristics of renal transplant recipients with upper gastrointestinal bleeding between endoscopic and non-endoscopic group.

| Characteristics |  | Endoscopy (n=33) | Non-endoscopy (n=22) | *p*-value |
| --- | --- | --- | --- | --- |
| Sex, n(%) | Male | 25(75.8%) | 16(72.7%) | 0.80 |
|  | Female | 8(24.2%) | 6(27.3%) |  |
| Age, average (±SD) |  | 46.9±11.4 | 47.9±9.1 | 0.73 |
| Anticoagulant drugs, n(%) | Negative | 30(90.9%) | 19(86.4%) | 0.60 |
| Intravenous hormone usage, n(%) | Negative | 24(72.7%) | 12(54.5%) | 0.17 |
| Calcineurin inhibitors, n(%) | Tacrolimus | 26(78.8%) | 21(95.5%) | 0.06 |
|  | Cyclosporine A | 7(21.2%) | 1(4.5%) |  |
| Diabetes, n(%) | Negative | 30(90.9%) | 17(77.3%) | 0.16 |
| Hepatitis（HBV/HCV), n(%) | Negative | 27(81.8%) | 16(72.7%) | 0.43 |
| Pulmonary infection, n(%) | Negative | 24(72.7%) | 10(45.5%) | 0.05 |
| Cardio-cerebrovascular disease, n(%) | Negative | 30(90.9%) | 18(81.8%) | 0.33 |
| Malignant tumor, n | Negative | 31(93.9%) | 21(95.5%) | 0.81 |
| Outcome, n(%) | Mortality | 5(15.2%) | 11(50.0%) | <0.01 |

Abbreviations：HBV: hepatitis B virus; HCV: hepatitis C virus.

Supplementary Table 2. Optimal cut-off values of each scoring system in predicting patients who might not need intervention.

| Scoring system | Cut-off | Number of low- risk patients（n,n%） | Sensitivity (%) | Spencificity (%) | PPV (%) | NPV (%) | Received transfusion  （n,n%） | Urgent intervention  （n,n%） | Mortality  （n,n%） |
| --- | --- | --- | --- | --- | --- | --- | --- | --- | --- |
| Glasgow Blatchford score | ≤6 | 10 (18.2%） | 45.0 | 97.1 | 75.6 | 90.0 | 1(1.8%) | 0.0 | 0.0 |
| AIMS65 score | ≤0 | 43 (78.2%） | 90.0 | 28.6 | 83.3 | 41.9 | 26(47.3%) | 5(9.1%) | 8(14.5%) |
| Pre-endoscopy Rockall score | ≤0 | 23 (41.8%） | 55.0 | 65.7 | 71.9 | 47.8 | 10(18.2%) | 3(5.5%) | 2(3.6%) |

PPV=positive predictive value; NPV=negative predictive value.

Mortality: Number (%) of patients dying within 90 days from presentation among patients classified as low risk.

Supplementary Table 3. Optimal thresholds to predict 90-day mortality in renal transplant recipients with upper gastrointestinal bleeding.

| Scoring system | Cut-off | No (%) of patients classified as high risk | Sensitivity (%) | Specificity (%) | PPV (%) | NPV (%) |
| --- | --- | --- | --- | --- | --- | --- |
| Glasgow Blatchford score | ≥14 | 17 (30.9%) | 68.8 | 84.6 | 64.7 | 86.8 |
| AIMS65 score | ≥1 | 12(21.8 %) | 56.2 | 92.3 | 75.0 | 83.7 |
| Pre-endoscopy Rockall score | ≥1 | 33 (58.2%) | 93.7 | 56.4 | 46.9 | 95.6 |

PPV=positive predictive value; NPV=negative predictive value.


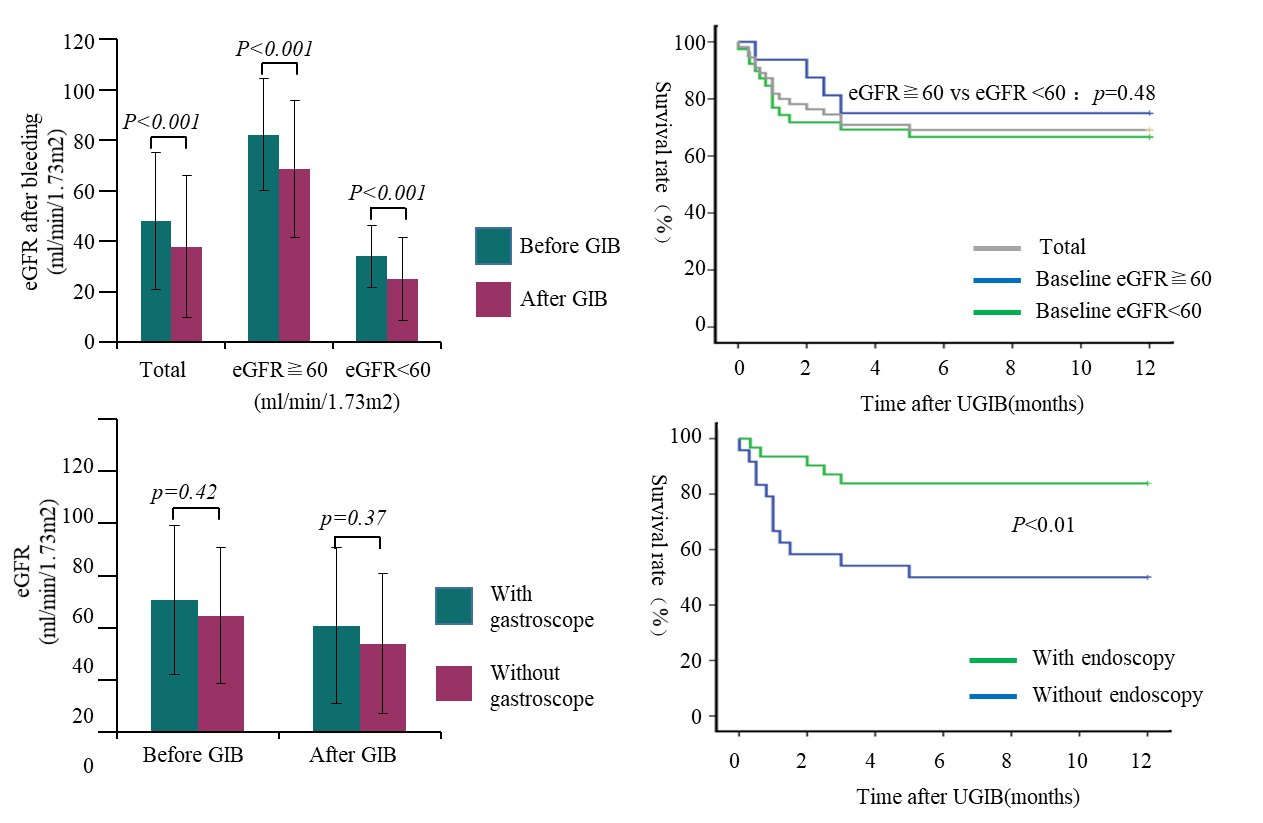


Supplementary figure 1. The survival rate and renal function of kidney transplant recipients with upper gastrointestinal bleeding.


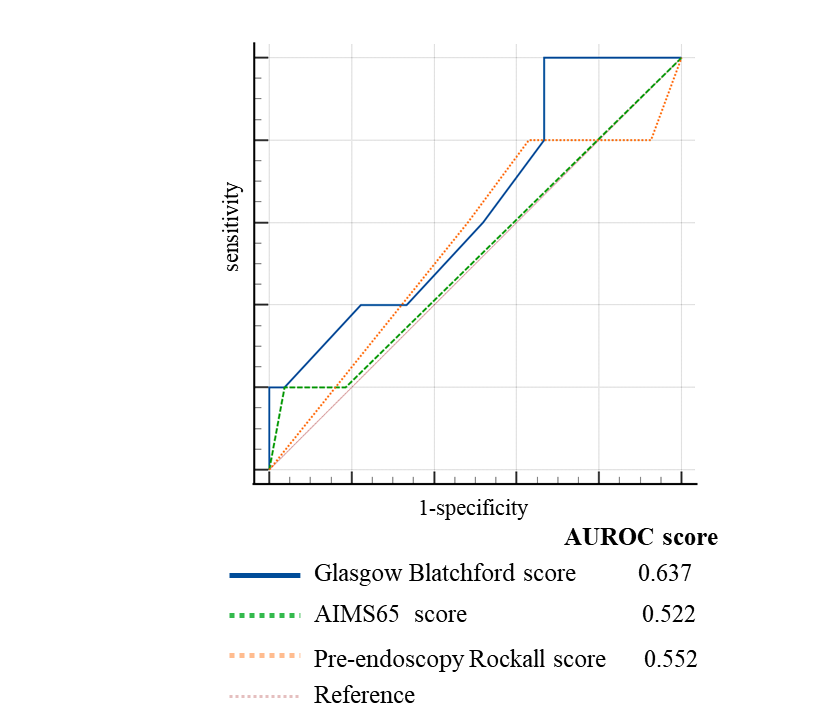


Supplementary figure 2. Comparison of three pre-endoscopy scoring systems on predicting need for urgent endoscopic intervention. AUROC: area under the receiver operating characteristic curve.
